# Demography, disorders and mortality of pet hamsters under primary veterinary care in the United Kingdom in 2016

**DOI:** 10.1111/jsap.13527

**Published:** 2022-06-22

**Authors:** D. G. O'Neill, K. Kim, D. C. Brodbelt, D. B. Church, C. Pegram, V. Baldrey

**Affiliations:** ^1^ Pathobiology and Population Sciences, The Royal Veterinary College Hatfield AL9 7TA UK; ^2^ Clinical Science and Services The Royal Veterinary College Hatfield AL9 7TA UK

## Abstract

**Background:**

Hamsters are popular pets worldwide but there is limited evidence on the overall health issues of pet hamsters. This study aimed to characterise the demography, disorder prevalence and mortality of pet hamsters in the United Kingdom.

**Method:**

The VetCompass study included anonymised clinical records of 16,605 hamsters.

**Results:**

The most common hamster species were Syrian (golden) (*Mesocricetus auratus*) (n=12,197, 73.45%), Djungarian (winter white dwarf) (*Phodopus sungorus*) (2286, 13.77%) and Roborovski hamsters (*Phodopus roborovskii*) (1054, 6.35%). The most prevalent precise‐level disorders recorded across all hamsters were a presentation categorised as ‘wet tail’ (n=293, 7.33%), disorder undiagnosed (292, 7.30%), bite injuries from other hamsters (235, 5.88%), overgrown nail(s) (165, 4.13%), overgrown incisor(s) (159, 3.98%) and traumatic injury (152, 3.80%). The most prevalent disorders groups across all species of hamster were traumatic injury (n=616, 15.41%), enteropathy (450, 11.26%), ophthalmological disorder (445, 11.13%), skin disorder (362, 9.05%) and mass (361, 9.03%). The median age at death across all hamsters was 1.75 years (interquartile range: 0.83 to 2.20, range: 0.01 to 3.65). The most common causes of death at a precise level were wet tail (7.88%, 95% confidence interval: 6.35 to 9.66), abdominal mass (6.40%, 95% confidence interval: 5.01 to 8.03), neoplasia (5.38%, 95% confidence interval: 4.11 to 6.90) and dyspnoea (3.99%, 95% confidence interval: 2.9 to 5.34).

**Conclusion:**

This study provides veterinary professionals, educators, welfare scientists and owners with an evidence base on pet hamster health. A greater understanding of the common disorders of pet hamsters can support veterinary professionals to communicate more effectively with owners on key issues and outcomes to expect from hamster ownership.

## BACKGROUND

Hamsters are rodents belonging to the subfamily *Cricetinae*, with the Syrian or golden hamster (*Mesocricetus auratus*) reportedly the most common and largest pet hamster species in the United Kingdom. The National Hamster Council additionally recognises four dwarf hamster species: Russian Campbell's (*Phodopus campbelli*), Djungarian or Russian winter white (*Phodopus sungorus*), Chinese (*Cricetulus griseus*) and Roborovski (*Phodopus roborovskii*) (National Hamster Council 2019). Hamsters have been kept as laboratory research subjects for decades and have become popular as companion animals, with an estimated 600,000 pet hamsters owned in the United Kingdom (Pet Food Manufacturers' Association 2021). However, there is limited published information on general health issues of pet hamsters (Adby & O'Neill 2004). The probability of presentation for veterinary care is lower among exotic pets such as hamsters than among more traditional companion species such as dogs and cats (Daviau 1999). Even when hamster species are presented for veterinary care, the limited evidence base on their common disorders, and often limited experience by veterinary professionals in handling hamsters, can often make delivery of high‐quality veterinary care challenging. In addition to their reputation for biting and being difficult to handle for clinical examination, hamsters are prey animals that have evolved to hide signs of illness therefore early recognition of problems is often challenging (Pellett & Mancinelli 2017a).

The evidence base on pet (companion) hamster disease is small (Nevarez 2011, Turner *et al*. 2017). Much of the published veterinary information on hamster health originates from hamsters in research settings and is often quite outdated. Furthermore, pet hamsters are reported to show very disparate disease presentations and prevalence from those described in laboratory colonies (Mans *et al*. 2020). A survey encompassing 87,880 hamsters from US‐based animal research breeding organisations reported wet tail, pneumonia, and neoplasia as the most common and most important naturally occurring diseases (Renshaw *et al*. 1975). However, pet hamsters are exposed to a much wider range of environmental conditions and stressors compared with the carefully controlled lives of research animals, with consequently differing frequencies of pathologies and physiological responses to be expected in pet hamsters (Mans *et al*. 2020). Much of the clinical literature that does exist on pet hamsters is based on individual case studies (Mangkoewidjojo & Kim 1977, Martorell *et al*. 2005, Fiskett 2011). A small number of retrospective studies are available, for example, reporting the incidence of neoplasia in hamsters (Kondo *et al*. 2008, Rother *et al*. 2021), but there is no literature examining overall disease prevalence in the UK pet hamster population.

This VetCompass study aimed to reduce these data gaps by reporting the demography, commonly diagnosed disorders and causes of mortality of pet hamsters under primary veterinary care in the United Kingdom (VetCompass 2022). The study was additionally specifically interested in comparing the health of Syrian (golden) hamsters with the health of other hamster species. The results from the current study could provide an evidence base for disorder prioritisation based on prevalence and assist veterinary professionals and owners in better understanding the health and welfare challenges and opportunities for pet hamsters. It is worth noting that this practice‐based research study reports the prevalence of disorders as diagnosed and reported in primary‐care practice. In some cases, the clinical presentations ascribed to certain diagnostic terms in primary‐care practice may differ from the meaning given to that same term in pathology or specialist literature. For example, the term ‘wet tail’ is frequently used as a syndromic term in primary‐care practice describing perineal soiling in hamsters from multiple sources including diarrhoea or discharges from the urinary tract or reproductive tract whereas the specialist literature generally retains the term ‘wet tail’ to refer to proliferative ileitis, caused by *Lawsonia intracellularis* (Turner *et al*. 2017, Pellett & Mancinelli 2017b, Baldrey 2021).

## METHODS

The study included 16,605 hamsters under primary veterinary care within VetCompass in 2016. Being ‘under veterinary care’ required (1) at least 1 electronic patient record (EPR) during 2016 (bodyweight, free‐text clinical note, treatment, VeNom diagnosis term) or (2) at least 1 EPR during both 2015 and 2017. VetCompass is a research programme that shares anonymized clinical records from primary‐care veterinary practices in the United Kingdom (VetCompass 2022). These shared data fields include a unique animal ID with associated metadata on species, breed, sex, neuter, date of birth and bodyweight, along with free‐form text clinical notes and summary diagnosis terms (VeNom Coding Group 2022) and treatments with associated dates. The design and analytic plans for the current study were deliberately aligned with previous VetCompass species‐based studies to facilitate reliable comparisons of common disorders between species (O'Neill *et al*. 2014, 2019a, 2021b).

A cohort study design was used to estimate the lifetime prevalence of the most commonly diagnosed disorders in hamsters under veterinary care during 2016. Power calculations showed that a sample of 3,523 hamsters was needed to estimate the prevalence of a disorder occurring in 3.0% of hamsters to a 0.5% margin of error (Epi Info, Centers for Disease Control and Prevention 2021). Ethics approval was obtained from the Royal Veterinary College Ethics and Welfare Committee (reference number SR2018‐1652).

All available clinical records from a random subset of hamsters were manually reviewed and all disorder events in the cohort data were followed over time to determine the most definitive diagnosis term recorded, as previously described (O'Neill *et al*. 2021b). Incident and pre‐existing presentations were not differentiated. Recurring ongoing conditions (e.g. dental overgrowth) were recorded only once. Clinical conditions that were not recorded with a biomedical diagnostic term were extracted using the first recorded presenting sign term (e.g. ‘lethargy’) as previously described (O'Neill *et al*. 2021b). Mortality data were extracted for all deaths recorded at any date and included the medical cause, date, the mechanism (euthanasia, unassisted death, unrecorded) and method of body disposal. The full lists of diagnosis terms were mapped to both precise and grouped levels of diagnostic precision as described previously (O'Neill *et al*. 2021b). Precise‐level terms provided disorder information to the highest level of diagnostic precision available within the clinical notes (e.g. *cystitis* would remain as *cystitis*) while disorder groups provided information at a more general level of diagnostic precision (e.g. *cystitis* would map to *urinary system disorder*).

Data checking and cleaning used Excel (Microsoft Office Excel 2013; Microsoft Corp.) and analysis used Stata Version 16 (Stata Corporation). The species information recorded in the EPR was mapped to a derived VetCompass list of hamster species terms (National Hamster Council 2015). Adult bodyweight (g) described the mean of all recorded bodyweights recorded at any date over 3 months of age for each hamster. Descriptive results for hamsters under veterinary care in 2016 were reported for species, sex, neuter status and adult (>3 months) bodyweight. Lifetime period prevalence values described the probability of diagnosis on at least one occasion for that disorder at any date during the available lifetime clinical records for each hamster. The 95% confidence intervals (CIs) estimates were calculated from standard errors based on an approximation to the binomial distribution (Kirkwood & Sterne 2003). Prevalence values were reported for hamsters overall and separately for Syrian (golden) hamsters. The Mann‐Whitney U test and Kruskal–Wallis test with *post hoc* testing was used to compare continuous variables as appropriate (Kirkwood & Sterne 2003). Multi‐variable binary logistic regression modelling was used to estimate the relative disorder odds for Syrian (golden) hamsters *versus* other hamster species. A separate model was built for each disorder that included a standard bank of covariables to account for confounding factors as previously described (hamster species and sex) (Dohoo *et al*. 2009, O'Neill *et al*. 2021b). The authors applied an ‘information theory’ approach to decide on which covariables to include in these standard models (Burnham & Anderson 2004). Results were reported from each regression model for only the association with the species of hamster (of a priori interest). Statistical significance was set at the 5% level.

## RESULTS

### Demography

The study population included 16,605 hamsters under primary veterinary care at 886 veterinary clinics during 2016. The most common hamster species were Syrian (golden) (*M. auratus*) (n=12,197, 73.45%), Djungarian (winter white dwarf) (*P. sungorus*) (n=2286, 13.77%) and Roborovski hamsters (*P. roborovskii*) (n=1054, 6.35%) (Table 1). The Syrian (golden) hamster [median bodyweight: 133 g, interquartile range (IQR): 100 to 160] weighed substantially heavier than the other common types of hamsters (median bodyweights varied between 25 and 46 g) (Table 1). There were 1487 (8.96%) hamsters that did not have sex recorded. Of the remainder, 7658 (50.65%) were female and 7460 (49.35%) were male. Overall, there were 90 (0.54%) hamsters recorded as neutered, including 43 (0.56%) females and 47 (0.63%) males. The median bodyweight across all hamster species did not differ between females (120 g, IQR: 62 to 150) and males (120 g, 56 to 152) (P=0.569).

**Table 1:** Counts and bodyweights of hamster species under primary veterinary care at practices participating in the VetCompass™ Programme in the United Kingdom from January 1 to December 31, 2016

Hamster species	Freq.	Percent	Median (IQR) adult bodyweight, g
Syrian (golden) hamster (*Mesocricetus auratus*)	12,197	73.45	133 (100 to 160)
Djungarian – winter white dwarf – Russian dwarf hamster (*Phodopus sungorus*)	2286	13.77	45 (34.5 to 58)
Roborovski hamster (*Phodopus roborovskii*)	1054	6.35	25 (20 to 30)
Dwarf hamster (species not specified)	824	4.96	45 (30 to 54)
Chinese dwarf hamster (*Cricetulus griseus*)	163	0.98	40 (31.5 to 48)
Campbell Russian hamster (*Phodopus cambelli*)	52	0.31	46 (38 to 50)
Other	29	0.18	

n=16,605

IQR Interquartile range

### Disorder prevalence

The EPRs of a random sample of 3998/16,605 (24.08%) hamsters were manually examined to extract information for each hamster on all recorded disorders at any date in the available clinical records. The median lifetime disorder count per hamster was one disorder (IQR: 1 to 2, range: 0 to 8). Among the three most common species, there was statistical evidence from *post hoc* testing that the median disorder count was higher in the Syrian (golden) hamster (n=2953, median: 1, IQR: 1 to 2, range: 1 to 7) than for either the Djungarian hamster (n=537, median: 1, IQR: 1 to 1, range: 1 to 8) or Roborovski hamster (n=269, median: 1, IQR: 1 to 1, range: 0 to 6) (Kruskal–Wallis P<0.001). The median disorder count did not vary between females (n=1829, median: 1, IQR: 1 to 2, range: 1 to 7) and males (n=1819, median: 1, IQR: 1 to 2, range: 0 to 8) (P=0.579).

At least one disorder was recorded for 3521 of 3998 (88.07%) hamsters. The remaining 11.93% did not have any disorder recorded. There was no evidence that the probability of having at least one disorder recorded differed between three most common species: Syrian (golden) (2615/2953, 88.55%), Djungarian (461/537, 85.85%) or Roborovski hamster (236/269, 87.73%) (P=0.200). There was weak evidence that female hamsters had a higher probability of having at least one disorder recorded than males (1625/1829, 88.85% *versus* 1578/1819, 86.75%, respectively) (P=0.053).

There were 5130 unique disorder events recorded across the 3998 hamsters, spanning 365 separate precise‐level disorder terms. The most prevalent precise‐level disorders recorded across all hamsters and also specifically in Syrian (golden) hamsters are shown in Table 2.

**Table 2:** Prevalence of the most common disorders at a *precise level of diagnostic precision* was recorded in all hamsters (n=3998) and Syrian (golden) hamsters (n=2954) under primary‐care veterinary care at UK practices participating in the VetCompass™ Programme from January 1 to December 31, 2016

Precise‐level term	All hamsters (n = 3998)			Syrian hamsters (n = 2954)		
	No.	%	95% CI	No.	%	95% CI
Wet tail*	293	7.33	6.54 to 8.18	268	9.08	8.06 to 10.17
Disorder not diagnosed	292	7.30	6.51 to 8.15	236	7.99	7.04 to 9.03
Bite injuries from other hamsters	235	5.88	5.17 to 6.65	89	3.01	2.43 to 3.70
Overgrown nail(s)	165	4.13	3.53 to 4.79	124	4.20	3.50 to 4.99
Overgrown incisor(s)	159	3.98	3.40 to 4.63	135	4.57	3.85 to 5.39
Traumatic injury	152	3.80	3.22 to 4.44	117	3.96	3.29 to 4.73
Abdominal mass	123	3.08	2.56 to 3.66	108	3.66	3.01 to 4.40
Conjunctivitis	122	3.05	2.54 to 3.63	92	3.12	2.52 to 3.81
Ocular discharge	113	2.83	2.33 to 3.39	98	3.32	2.70 to 4.03
Diarrhoea	112	2.80	2.31 to 3.36	95	3.22	2.61 to 3.92
Wound	110	2.75	2.27 to 3.33	66	2.24	1.73 to 2.83
Mange	106	2.65	2.18 to 3.20	82	2.78	2.21 to 3.44
Abscess	98	2.45	2.00 to 2.98	71	2.40	1.88 to 3.02
Alopecia	85	2.13	1.70 to 2.62	69	2.34	1.82 to 2.95
Skin lesions	79	1.98	1.57 to 2.46	55	1.86	1.41 to 2.42
Upper respiratory tract infection	78	1.95	1.55 to 2.43	66	2.24	1.73 to 2.83
Anorexia	74	1.85	1.46 to 2.32	65	2.20	1.70 to 2.80
Neoplasia	74	1.85	1.46 to 2.32	65	2.20	1.70 to 2.80
Bite injury	70	1.75	1.37 to 2.21	20	0.68	0.41 to 1.04
Dyspnoea	65	1.63	1.26 to 2.07	49	1.66	1.23 to 2.19

CI Confidence interval

* Wet tail in a primary‐care veterinary context covers multiple forms of perineal soiling including diarrhoea or discharges from the urinary tract or reproductive tract

There were 50 distinct disorder groups recorded. The most prevalent disorder groups across all hamsters and also specifically in Syrian (golden) hamsters are shown in Table 3. After taking sex into account in multi‐variable modelling, Syrian (golden) hamsters had higher odds of seven of 20 (35%) common disorder groups and lower odds of one of 20 (5%) common disorder groups compared with hamsters that were not Syrian (golden) hamsters. Disorder groups with the highest odds ratio for Syrian (golden) compared with other hamsters included female reproductive disorder [odds ratio (OR): 5.19, 95% CI: 2.09 to 12.87, P<0.001] and urinary system disorder (OR: 5.04, 95% CI: 2.33 to 10.88, P<0.001). The single disorder group with lower odds in Syrian (golden) compared with other hamsters was traumatic injury (OR: 0.34, 95% CI: 0.28 to 0.40, P<0.001) (Table 3).

**Table 3:** Prevalence of the most common disorders at a *grouped level of diagnostic precision* was recorded in all hamsters (n=3998) and Syrian (golden) hamsters (n=2954) under primary‐care veterinary care at UK practices participating in the VetCompass™ Programme from January 1 to December 31, 2016

Grouped‐level term	All hamsters (n = 3998)		Syrian (Golden) hamsters (n = 2954)		Odds ratio	95% CI	P value
	No.	Prevalence (95% CI)	No.	Prevalence (95% CI)			
Traumatic injury	616	15.41 (14.30 to 16.56)	327	11.07 (9.96 to 12.26)	0.34	0.28 to 0.40	<0.001
Enteropathy	450	11.26 (10.29 to 12.28)	404	13.68 (12.46 to 14.97)	3.54	2.58 to 4.86	<0.001
Ophthalmological disorder	445	11.13 (10.17 to 12.15)	340	11.51 (10.38 to 12.72)	1.17	0.92 to 1.47	0.196
Skin disorder	362	9.05 (8.18 to 9.99)	264	8.94 (7.94 to 10.03)	0.93	0.73 to 1.18	0.542
Mass	361	9.03 (8.16 to 9.96)	269	9.11 (8.10 to 10.21)	1.02	0.79 to 1.31	0.883
Disorder not diagnosed	292	7.30 (6.52 to 8.15)	236	7.99 (7.04 to 9.03)	1.50	1.11 to 2.03	0.008
Neoplasia	277	6.93 (6.16 to 7.76)	198	6.71 (5.83 to 7.67)	0.87	0.67 to 1.15	0.334
Dental disorder	197	4.93 (4.28 to 5.64)	170	5.76 (4.94 to 6.66)	2.27	1.50 to 3.43	<0.001
Parasite infestation	174	4.35 (3.74 to 5.03)	135	4.57 (3.85 to 5.39)	1.28	0.89 to 1.85	0.181
Claw/nail disorder	169	4.23 (3.62 to 4.90)	127	4.3 (3.60 to 5.10)	1.11	0.78 to 1.59	0.565
Upper respiratory tract disorder	133	3.33 (2.79 to 3.93)	112	3.79 (3.13 to 4.55)	1.89	1.18 to 3.03	0.008
Lower respiratory tract disorder	119	2.98 (2.47 to 3.55)	86	2.91 (2.34 to 3.58)	0.90	0.60 to 1.36	0.616
Thin	108	2.70 (2.22 to 3.25)	82	2.78 (2.21 to 3.44)	1.13	0.72 to 1.77	0.598
Urinary system disorder	108	2.70 (2.22 to 3.25)	101	3.42 (2.79 to 4.14)	5.04	2.33 to 10.88	<0.001
Female reproductive disorder*	102	2.55 (2.08 to 3.09)	95	3.22 (2.61 to 3.92)	5.19	2.09 to 12.87	<0.001
Abscess	98	2.45 (1.99 to 2.98)	71	2.4 (1.88 to 3.02)	0.95	0.61 to 1.49	0.831
Collapsed	87	2.18 (1.75 to 2.68)	71	2.4 (1.88 to 3.02)	1.58	0.91 to 2.73	0.103
Lethargy	82	2.05 (1.63 to 2.54)	63	2.13 (1.64 to 2.72)	1.17	0.70 to 1.97	0.551
Brain disorder	78	1.95 (1.55 to 2.43)	61	2.07 (1.58 to 2.65)	1.24	0.72 to 2.14	0.44
Appetite disorder	74	1.85 (1.46 to 2.32)	65	2.2 (1.70 to 2.80)	2.68	1.33 to 5.41	0.006

The odds ratio reports the odds in Syrian (golden) hamsters compared with hamsters that were not Syrian (golden) hamsters

CI Confidence interval

* Analysis only included females

### Mortality

There were 1356 deaths recorded among the 3998 (33.92%) hamsters. Information on the age at death was available for 1055 (77.81%) deaths. The median age at death across all hamsters was1.75 years (IQR: 0.83 to 2.20, range: 0.01 to 3.65). The median age at death did not differ between Syrian (golden) (1.76 years, IQR: 0.91 to 2.20, 0.01 to 3.65) and non‐Syrian (golden) hamsters (1.68 years, IQR: 0.51 to 2.19, range: 0.08 to 3.62) (P=0.280). The median age at death in males (2.00 years, IQR: 1.00 to 2.39, range: 0.16 to 3.65) was higher than females (1.67, IQR: 0.93 to 2.04, 0.08 to 3.30) (P<0.001). The mechanism of death was recorded for 1304 (96.17%) deaths. Among deaths with the mechanism recorded, there were 1143 (84.29%) deaths by euthanasia and 161 (11.87) unassisted deaths. The median age at death did not differ significantly between deaths by euthanasia (1.75 years, 0.83 to 2.17, 0.01 to 3.65) and unassisted deaths (1.59 years, 0.61 to 2.23, 0.16 to 3.50) (P=0.267). The method of body disposal was recorded in 686 of 1356 (50.59%) deaths. Of those deaths with a method of disposal recorded, 385 (56.12%) were mass cremated, 80 (11.66%) were individually cremated and 221 (32.22%) were buried at home by the owners.

The cause of death was recorded for 1078 (79.50%) deaths. Among deaths with a recorded cause, the most common causes of death at a precise level across all hamsters and also specifically in Syrian (golden) hamsters are shown in Table 4. The median age at death across the most common causes of death at a precise level varied from 0.34 years (wet tail) to 2.11 years (skin neoplasm) (Table 4).

**Table 4:** Proportional mortality from the most common disorders at a *precise level of diagnostic precision* was recorded in all hamsters (n=1356) and Syrian (golden) hamsters (n=1099) under primary‐care veterinary care at UK practices participating in the VetCompass™ Programme from January 1 to December 31, 2016

Precise‐level term	All deaths (n = 1356)				Deaths with cause diagnosed		Syrian (golden) hamsters – Deaths with cause diagnosed		
	No.	%	95% CI	Median age at death (years)	%	95% CI	No.	%	95% CI
Disorder not diagnosed	278	20.50	18.38 to 22.75	2.00					
Wet tail*	85	6.27	5.04 to 7.69	0.34	7.88	6.35 to 9.66	75	8.56	6.79 to 10.61
Abdominal mass	69	5.09	3.98 to 6.40	1.51	6.40	5.01 to 8.03	62	7.08	5.47 to 8.98
Neoplasia	58	4.28	3.26 to 5.49	1.64	5.38	4.11 to 6.90	50	5.71	4.27 to 7.46
Dyspnoea	43	3.17	2.30 to 4.25	1.75	3.99	2.90 to 5.34	33	3.77	2.61 to 5.25
Collapsed	35	2.58	1.80 to 3.57	2.00	3.25	2.27 to 4.49	28	3.20	2.13 to 4.59
Traumatic injury	34	2.51	1.74 to 3.49	1.00	3.15	2.19 to 4.38	29	3.31	2.23 to 4.72
Moribund	29	2.14	1.44 to 3.06	1.64	2.69	1.81 to 3.84	22	2.51	1.58 to 3.78
Pyometra	28	2.06	1.38 to 2.97	1.29	2.60	1.73 to 3.73	26	2.97	1.95 to 4.32
Diarrhoea	27	1.99	1.32 to 2.88	2.00	2.50	1.66 to 3.62	25	2.85	1.86 to 4.18
Anorexia	26	1.92	1.26 to 2.80	2.00	2.41	1.58 to 3.51	23	2.63	1.67 to 3.91
Skin neoplasm	24	1.77	1.14 to 2.62	2.11	2.23	1.43 to 3.29	15	1.71	0.96 to 2.81
Paresis/paralysis	23	1.70	1.08 to 2.53	1.44	2.13	1.36 to 3.18	18	2.05	1.22 to 3.23
Skin mass	20	1.47	0.90 to 2.27	1.80	1.86	1.14 to 2.85	17	1.94	1.13 to 3.09
Anal disorder	19	1.40	0.85 to 2.18	1.00	1.76	1.06 to 2.74	16	1.83	1.05 to 2.95
Ataxia	18	1.33	0.79 to 2.09	2.00	1.67	0.99 to 2.63	12	1.37	0.71 to 2.38

CI Confidence interval

* Wet tail in a primary‐care veterinary context covers multiple forms of perineal soiling including diarrhoea or discharges from the urinary tract or reproductive tract

The most common causes of death at a grouped level of precision across all hamsters and also specifically in Syrian (golden) hamsters are shown in Table 5. The median age at death across the most common causes of death at a grouped level of precision varied from 0.50 years (enteropathy) to 2.00 years (mass, collapsed and central nervous system disorder) (Table 5).

**Table 5:** Proportional mortality from the most common disorders at a *grouped level of diagnostic precision* with a cause of death was recorded in all hamsters (n=1078) and Syrian (golden) hamsters (n=876) under primary‐care veterinary care at UK practices participating in the VetCompass™ Programme from January 1 to December 31, 2016

Grouped‐level term	All hamsters (n = 1078)			Syrian (golden) hamsters		Odds ratio	95% CI	P value
	No.	% (95% CI)	Median age at death (years)	No.	% (95% CI)			
Neoplasia	151	14.01 (11.99 to 16.22)	1.76	116	13.24 (11.07 to 15.67)	0.76	0.51 to 1.15	0.194
Mass	147	13.64 (11.64 to 15.83)	2.00	123	14.04 (11.81 to 16.52)	1.23	0.78 to 1.96	0.376
Enteropathy	127	11.78 (9.92 to 13.86)	0.50	113	12.90 (10.75 to 15.30)	2.03	1.13 to 3.63	**0.017**
Traumatic injury	79	7.33 (5.84 to 9.05)	1.00	53	6.05 (4.56 to 7.84)	0.48	0.29 to 0.79	**0.004**
Lower respiratory tract disorder	68	6.31 (4.93 to 7.93)	1.58	49	5.59 (4.17 to 7.33)	0.56	0.32 to 0.97	**0.039**
Collapsed	64	5.94 (4.60 to 7.52)	2.00	50	5.71 (4.27 to 7.46)	0.86	0.47 to 1.59	0.627
Spinal cord disorder	44	4.08 (2.98 to 5.44)	1.91	33	3.77 (2.61 to 5.25)	0.72	0.36 to 1.44	0.352
Ophthalmological disorder	43	3.99 (2.90 to 5.34)	1.18	37	4.22 (2.99 to 5.78)	1.41	0.58 to 3.38	0.446
Skin disorder	42	3.90 (2.82 to 5.23)	1.53	35	4.00 (2.80 to 5.51)	1.11	0.48 to 2.54	0.798
Central nervous system disorder	38	3.53 (2.51 to 4.81)	2.00	34	3.88 (2.7 to 5.38)	1.82	0.64 to 5.19	0.264

CI Confidence interval

## DISCUSSION

The Syrian (golden) hamster was the most commonly recorded species in the current study, accounting for 73.45% of the pet hamsters. This is consistent with earlier reports that Syrian hamsters were the most commonly owned pet species of hamster in the United Kingdom and suggests that this relative preference for Syrian compared with other hamster species is not waning over time (Keeble 2009). Based on the evidence from the current study, there seems to be little movement towards increasing demand for extreme sizes or conformations in hamsters in the United Kingdom, in contrast to the situation in dogs (Packer *et al*. 2019) and rabbits (Harvey *et al*. 2019). This is supported by the median bodyweight for Syrian hamsters (133 g) and dwarf hamster species (25 to 46 g) in our study remaining consistent with previously reported average weights of 110 to 140 g (Wilson *et al*. 2012) and 30 to 50 g (Bauer & Besch‐Williford 2012), respectively.

In the current study, just 0.54% of hamsters were recorded as neutered in contrast to dogs where over 40% of males and females are reported as neutered in the United Kingdom (O'Neill *et al*. 2021b). The cost:benefit of routine neutering for disease prevention in hamsters is not well‐documented. Indeed, in a laboratory setting, neutered male Syrian hamsters have an increased risk of atrial thrombosis and amyloidosis (Sichuk *et al*. 1965, Coe & Ross 1990), so neutering in that population may have been contraindicated. In addition, many hamsters are kept as solitary pets or in single‐sex groups, reducing potential benefits from neutering for population control (Capello 2003).

In the current study, 88.07% of hamsters were recorded with at least one disorder. This is higher than the 65.84% of UK dogs (O'Neill *et al*. 2021b) and 68.3% of UK cats (O'Neill *et al*. 2014) that are recorded annually with at least one disorder within ‘VetCompass’. [Corrections added on 12 July 2022 after first online publication: In the preceding sentence,” REDACTED FOR REVIEW” has been corrected to “VetCompass”.] Low levels of routine veterinary prophylactic care (e.g. elective neutering, microchipping for vaccination) for pet hamsters in the United Kingdom means that a high proportion of ‘unwell animal’ visits among the veterinary primary‐care caseload of hamsters is unsurprising (Royal Society for the Prevention of Cruelty to Animals 2022). That said, the proportion of hamsters presenting without a health problem (11.93%) was higher than previously suggested in the literature (Daviau 1999). Although the practices in the current study all offer first‐opinion veterinary care, some of the practices contributing to data for the current study are associated with pet retailers and this may have increased the proportion of hamsters benefitting from routine health checks, e.g. postpurchase checks. Further investigation would be needed to determine the true nature and levels of routine wellness visits for hamsters overall in the United Kingdom.

Slightly more females than males had at least one disorder recorded. There are some sex differences in disease prevalence reported in the literature. For example, female hamsters are reportedly predisposed to show amyloidosis more severely and at an earlier age than males (Brown & Donnelly 2012, Mans *et al*. 2020). In the current study, the health differences between the sexes were small, however, and unlikely to lead to major clinical significance.

Traumatic injury represented the most common disorder group in the current study, affecting 15.41% of hamsters, although Syrian hamsters had lower odds compared with other hamsters. This is consistent with a study reporting traumatic injuries as one of the most frequent causes of emergency veterinary presentation in rodents (Hawkins & Graham 2007) and highlights the welfare burden from these preventable incidents. Syrian hamsters are generally considered solitary pets, with fighting within group‐housed animals well‐documented (Murray 2012). However, dwarf species are more commonly kept in social groups (Lennox & Bauck 2012). Information on the housing methods (solitary or cohoused) of the hamsters was not available in the current study but elicitation of this information by the attending veterinarians could help to improve husbandry practices and reduce the risk of fighting with conspecifics. Other traumatic injuries may result from inappropriate handling, being dropped or stepped on, electrocution and attack by other household pets (Hawkins & Graham 2007, Pellett & Mancinelli 2017b). Many of these events are preventable and therefore represent an opportunity for enhanced owner education to improve animal welfare and health.

Enteropathy was the second most common disorder group reported across all species, affecting 11.26% of hamsters, and was the most common disorder group reported in Syrian hamsters, consistent with the literature (Lennox & Bauck 2012). Wet tail was the most common precise‐level disorder reported and this was also the most common cause of death. It is important to note that ‘wet tail’ technically refers to proliferative ileitis, caused by *L. intracellularis* (Baldrey 2021). However, ‘wet tail’ is frequently used in primary‐care veterinary practice as a syndromic term to more generally describe any form of diarrhoea in hamsters, regardless of cause (Pellett & Mancinelli 2017b) and is even sometimes extended to describe any perineal soiling by urinary tract or reproductive tract discharges. Further research, including postmortem and histopathology findings, would be required to determine the true prevalence of proliferative ileitis per se *versus* other aetiologies for diarrhoea and perineal soiling in hamsters.

Ophthalmological disorders were also commonly reported in the current study, affecting 11.13% of all hamsters. Conjunctivitis and ocular discharge were the most commonly reported ophthalmological precise‐level diagnoses. Conjunctivitis is reported to be common in pet hamsters (Montiani‐Ferreira 2009) and can indicate husbandry deficits related to dusty bedding as well as other ocular pathologies (Williams 2012).

Skin disorders were recorded in 9.05% of hamsters and included alopecia and unspecified skin lesions. Bite wounds, neoplasia, mange and secondary pyoderma are the most commonly reported skin lesions in the literature (Longley 2009, Mans *et al*. 2020), although poor coat condition can also be a non‐specific sign of ill health in hamsters.

Some of the commonly recorded disorders in the current study could suggest that husbandry deficits are common in pet hamsters. Overgrown nails were identified in 4.13% of pet hamsters. This is not commonly discussed in the literature relating to pet hamsters but has been linked with insufficient exercise or inappropriate bedding substrates in rabbits (O'Neill *et al*. 2019a). Incisor overgrowth was reported in 3.98% of hamsters and has also been linked to husbandry deficits leading to a deficiency of dental wear (Verstraete 2003). Providing enrichment in the form of cardboard and wooden items to gnaw on and the opportunity for exercise to wear the nails may help reduce this incidence.

Disorders with the highest odds ratio for Syrian compared with other hamsters included female reproductive disorders and urinary tract disorders. Pyometra has been described in Syrian hamsters but is reportedly uncommon (Pisu *et al*. 2012). Female hamsters typically show a creamy white vaginal discharge at the end of normal oestrus and this can easily be mistaken for a purulent discharge. Ultrasonographic examination of the reproductive tract or prolonged presence beyond 24 to 36 h of a creamy discharge can assist to differentiate normal oestrus from a true pyometra (Mans *et al*. 2020). Urinary tract disorders previously reported in Syrian hamsters include urolithiasis and amyloidosis (Capello 2002, Dutton 2020). These disorders may be truly over‐represented in Syrian hamsters *versus* other species or may just represent a diagnosis bias whereby these conditions are more easily recognised in the Syrian hamster due to the latter's larger body size.

It is noteworthy that 7.3% of the hamsters were recoded with a disorder that was not diagnosed, making ‘disorder not diagnosed’ the second most common precise‐level disorder recorded across all hamster species. Using a similar study design, there were just 0.81% of dogs recorded with ‘disorder not diagnosed’, making it the 39th most common precise‐level disorder. The high proportion of undiagnosed disorders in hamsters may reflect incomplete recording of the clinical records or could indicate low familiarity by veterinary surgeons with the common disease processes, clinical signs and diagnostics in hamsters. This finding of frequently undiagnosed disorders in hamsters may support calls for enhanced undergraduate and postgraduate training and teaching in exotic medicine (Fitzpatrick & Mellor 2003, Rosenthal 2006).

The current study reported a median age at death of 1.75 years across all hamsters under primary veterinary care. Knowledge of the typical ages at death of pet hamsters under veterinary care can help veterinarians to build realistic expectations for hamster owners and may also help owners to accept the need for euthanasia when recommended by their veterinary surgeon.

The ages at death under veterinary care did not differ between the common hamster species in our study. However, earlier ages at death of 0.75 to 1.25 years have previously been reported for some dwarf hamster species in laboratory settings (Wilson *et al*. 2012). In our study, males had a longer median age at death of 2 years compared with 1.67 years for females. This difference represents a relatively large proportion of the overall lifespan of hamsters and is consistent with the longer lifespan of males compared with female Syrian hamsters previously reported in a laboratory setting (McMartin 1979). It is widely recognised that the clinical priorities and care requirements change in older patients (Gardner 2017, Lennox 2020, Reavill & Imai 2020), and these changes may require earlier attention for female hamsters if they have shorter lifespans than males. Animal health organisations recommend more frequent examinations for dogs and cats when they reach geriatric age (Force *et al*. 2005, Pittari *et al*. 2009, Vogt *et al*. 2010), and encouraging similar routine geriatric visits for exotic pets may offer a valuable opportunity to educate owners on common geriatric conditions and changes to care, assess quality‐of‐life, and communicate expectations and options for end‐of‐life.

Cause of death was recorded for 79.5% of deaths, with a presentation categorised as ‘wet tail’ (6.27% of deaths) recorded as the most common cause of death. Other common causes of death included abdominal mass, neoplasia and dyspnoea. Studies of neoplasia in pet Djungarian and Syrian hamsters reported a mean age of affected individuals of 1.65 years (Kondo *et al*. 2008), and 1 year (Rother *et al*. 2021). Those studies reported a higher prevalence of neoplastic disease in Djungarian than Syrian hamsters, consistent with the laboratory literature. However, this contrasts with the results of the current study where the prevalence of neoplasia did not differ between the hamster species.

Dyspnoea was the fourth most common cause of mortality in pet hamsters in the current study, recorded in 3.99% of hamster deaths, but was not a common cause of morbidity in the living hamsters. Respiratory disease has been reported as rare in pet hamsters (Mans *et al*. 2020), but conversely as being a common cause of morbidity and mortality in laboratory hamsters (Renshaw *et al*. 1975). Cardiovascular disease, however, is common in pet hamsters, and in advanced cases can lead to dyspnoea, with a poor prognosis (Brown & Donnelly 2012, Mans *et al*. 2020). Further detail is needed to establish whether the dyspnoea cases in the current study were due primarily to respiratory disease or whether instead there was also cardiac involvement. Greater understanding here could inform improved treatment options.

Euthanasia was the most common means of death for hamsters under veterinary care in the current study, accounting for 84.29% of hamster deaths compared with 11.87% of unassisted deaths. Unassisted deaths in the overall national hamster population are likely to be under‐represented in the current study, as many hamsters may not have been presented for veterinary care or have had their death reported to the veterinary surgery if they died at home. Illness and deterioration can be challenging for owners to recognise in pet rodents and therefore hamsters are commonly presented late in the course of disease (Mans *et al*. 2020). Consequently, euthanasia may often be elected on welfare grounds, due to poor prognosis by then.

This study had some limitations as previously reported (O'Neill *et al*. 2021b). The clinical data were sourced from primary‐care practices participating in the VetCompass Programme, which may not be representative of all primary‐care clinics in the United Kingdom. Of the hamsters with at least one precise‐level disorder recorded, 8.4% of disorders were ‘undiagnosed’ so some conditions may be under‐represented in the study. Many disorders and causes of death are descriptions of the dominant clinical signs (e.g. dyspnoea) or reflect common‐use diagnostic terms (e.g. wet tail) rather than conforming with formal scientific biomedical diagnostic terminology that may be more commonly used in biomedical research and teaching (Bodenreider *et al*. 2004). Biomedical diagnosis terms represent invariant features of biomedical reality whereas the more clinically descriptive names convey how this reality is perceived, measured and understood by veterinary professionals. In the current study, we did not aim to artificially replace commonly used descriptive terms such as wet tail with our choice of biomedical replacement terms such as enteropathy and consequently we hope that the results we report are a truer reflection of care that is delivered in UK primary veterinary care. Precise‐level disorders can be grouped in several different ways, including by body system, organ affected and pathophysiology. Different grouping methods to that used in the current study may have led to numerically different results for the grouped disorders. The current study reported on lifetime disorder prevalence as an overall measure of disorder burden in hamsters, in line with similar methods used to report 1‐year disorder prevalence in dogs overall or by breed (O'Neill *et al*. 2019b, 2021b). The study did not aim to report disorder incidence or report the ages at first diagnosis for each disorder but this could be the subject of future studies, in line with previous disorder‐specific studies in dogs (O'Neill *et al*. 2021a, Schofield *et al*. 2021). The study included only hamsters presented to veterinary practices, so the conclusions may not apply to all pet hamsters in the United Kingdom. However, taken in combination with the findings of other studies in the literature, the current study should contribute to an improved understanding of common diseases in the pet hamster species.

This study represents the largest veterinary practice‐based study of hamster demography, disorder and mortality in the United Kingdom. It adds to a previously limited evidence base on pet hamster health and provides veterinary professionals with further information on the most common health conditions and typically expected ages at death for pet hamsters presenting to primary‐care veterinary practices in the United Kingdom. This can help guide further research and education to improve the health and welfare of pet hamsters.

## Conflict of interest

None of the authors of this article has a financial or personal relationship with other people or organisations that could inappropriately influence or bias the content of the paper

## Author contributions

DON and KK were responsible for the conception and design of the study and the acquisition and extraction of data. DON carried out the analysis. DON, VB and KK were mainly responsible for drafting the manuscript. DON, VB, KK, DC, DB and CP were involved in interpreting the results, revising the manuscript and giving final approval of the version to be published. DON, VB, KK, DC, DB and CP agree to be accountable for all aspects of the accuracy and integrity of the work. All authors have read and approved the manuscript.

## Ethics statement

Ethics approval was granted by the Royal Veterinary College ‘Ethics and Welfare Committee’. The reference number was SR2018‐1652.
